# The national employment guarantee scheme and inequities in household spending on food and non-food determinants of health in rural India

**DOI:** 10.1186/1475-9276-12-84

**Published:** 2013-10-15

**Authors:** TR Dilip, Rakhi Dandona, Lalit Dandona

**Affiliations:** 1 Public Health Foundation of India, ISID Campus, 4 Institutional Area, Vasant Kunj, New Delhi 110070, India; 2 Institute for Health Metrics and Evaluation, University of Washington, 2301 Fifth Avenue, Suite 600, Seattle, WA 98103, USA

**Keywords:** Consumption expenditure, Employment scheme, Food, Health, India, Inequity

## Abstract

**Introduction:**

Inequities in a population in spending on food and non-food items can contribute to disparities in health status. The Mahatma Gandhi National Rural Employment Guarantee Scheme (MGNREGS) was launched in rural India in 2006, aimed at providing at least 100 days of manual work to a member in needy households.

**Methods:**

We used nationally representative data from the consumer expenditure surveys of 2004–05 and 2009–10 and the employment survey of 2009–10 conducted by National Sample Survey Organisation to assess the effect of MGNREGS in reducing inequities in consumption of food and non-food items between poor and non-poor households in the states of India. Variations among the states in implementation of MGNREGS were examined using the employment and unemployment survey data, and compared with official programme data up to 2012–13. Inequity in spending on food and non-food items was assessed using the ratio of monthly per capita consumer expenditure (MPCE) between the most vulnerable (labourer) and least vulnerable categories of households.

**Results:**

The survey data suggested 1.42 billion person-days of MGNRGES employment in the 2009–10 financial year, whereas the official programme data reported 2.84 billion person-days. According to the official data, the person-days of MGNRGES employment decreased by 43.3% from 2009–10 to 2012–13 for the 9 large less developed states of India. Survey data revealed that the average number of MGNREGS work days in a year per household varied from 42 days in Rajasthan to less than 10 days in 14 of the 20 major states in India in 2009–10. Rajasthan with the highest implementation of MGNRGES among the 9 less developed states of India had the highest relative decline of 10.4% in the food spending inequity from 2004–05 to 2009–10 between the most vulnerable and less vulnerable households. The changes in inequity for non-food spending did not have any particular pattern across the less developed states. In the most vulnerable category, the households in Rajasthan that got 100 or more days of work in a year under MGNREGS had a 25.9% increase in MPCE.

**Conclusion:**

MGNREGS seems to have contributed to the reduction in food consumption inequity in rural Rajasthan in 2009–10, and has the potential of making a similar contribution with higher level of implementation of this programme in other states. Non-food consumption inequities benefited less from MGNRGES until 2009–10. The reported decrease in the MGNRGES employment person-days in the less developed states of India from 2009–10 to 2012–13 is of concern.

## Background

Disparities in health status among sub-groups in a population are influenced by broader determinants, commonly referred to as social determinants of health [1]. Accordingly, reduction in differences in basic living conditions between different population groups in a country could contribute to reduction of health inequalities. The Government of India legislated the Mahatma Gandhi National Rural Employment Guarantee Act in 2005 which guarantees at least 100 days of wage employment in a financial year to every household where an adult member volunteers to do unskilled manual work [2]. The Mahatma Gandhi National Employment Guarantee Scheme (MGNREGS) was started in a phased manner in 2006 and is now operational in rural areas of all districts in India since April 2008. According to the official data, this social protection scheme provided 2.25 billion days of employment to about 50 million households in the year 2012–13 with an estimated expenditure of Indian Rupees 393 billion (US$ 7.2 billion) [3]. The level of participation in MGNREGS has been noted to be relatively higher among the poor and the socially vulnerable sections of the population as this scheme offers unskilled manual labour employment [4,5].

Previous studies have suggested that in addition to directly increasing household income of the beneficiary, MGNREGS may also be contributing to improving working conditions in labour market through increasing the bargaining power of poor men and women, reducing temporary migration for work from rural areas, and reducing wage gaps between males and females [5-10]. It has also been suggested that this employment scheme has contributed to improvements in schooling of girl children in the beneficiary households [9]. The potential of MGNREGS to contribute to improving food and non-food consumption that could reduce health inequities in the population has not yet been reported. In this paper we report this analysis using data from the national employment and consumer expenditure surveys conducted by the National Sample Survey Organisation of India.

## Methods

### Data

We used data from the following nationwide surveys of the National Sample Survey Organisation (NSSO): consumer expenditure surveys of 2004–05 and 2009–10, and the employment and unemployment survey of 2009–10 [4,11,12]. The consumer expenditure survey of 2004–05 represents the pre-MGNREGS period and the 2009–10 consumer survey represents the post-MGNREGS period. These surveys used stratified multistage sampling of households with the aim of having the sample representative of each state of India. The 2004–05 consumer expenditure survey had a sample of 79,298 rural households in India and the 2009–10 survey had a sample of 59,097 rural households. These consumer surveys provide data on expenditure by each sampled household and the break-up of this expenditure by several categories. Food expenditure is available by cereals, pulses, milk and milk products, sugar, salt, edible oil, egg, fish and meat, vegetables, fresh fruits, dry fruits, spices and beverages. Non-food items include expenses on pan, tobacco, intoxicants, fuel and light, clothing, bedding, footwear education, medical (inpatient and outpatient separately), entertainment, minor durable type goods, toilet articles, other household consumables, consumer services, conveyance, rent, consumer taxes and cess, and purchase and construction (including maintenance and repair) of various durable goods for domestic use. The reference period for collection of consumption expenditure data from households was 365 days for education, inpatient treatment, clothing, bedding, footwear and durable goods. For all other items the reference period was 30 days.

The 2009–10 consumer expenditure survey did not ask whether the household participated in MGNREGS. Data from NSSO’s nationwide employment and unemployment survey of 2009–10, which included 59,128 rural households and documented participation in MGNREGS, were used to study the differentials in consumption of food and non-food items between beneficiaries of this scheme and the non-beneficiaries. This survey provides data on whether households registered for MGNREGS, the number of days of work received in a year by a household under MGNREGS and details of the consumption expenditure by each household. We compared the person-days of employment due to MGNREGS estimated from the employment and unemployment survey of 2009–10 with those reported by the official programme data [3], and the change from 2009–10 to 2012–13 reported by the official data.

The NSSO surveys classify sample households based on its major source of income (that contributes more than 50% of household income) in these categories [4]: labourer agriculture, labourer non-agriculture, self-employed agriculture, self-employed non-agriculture, others. Based on the anticipated economic vulnerability of these categories, we considered these three categories for our analysis representing the highest to the lowest vulnerability in this order: labourer (agriculture and non-agriculture), self-employed agriculture, and others (self-employed non-agriculture and others).

### Assessment of inequities

Inequity was defined as the ratio of household monthly per capita expenditure (MPCE) between the least and most vulnerable occupation categories (others and labourers, respectively). The impact of MGNREGS in reducing inequities in the household monthly per capita expenditure was examined for all commodities together, all food items, and all non-food items. In addition, the impact was assessed separately for the cereal and non-cereal food items, as cereals are relatively less expensive basic food whereas non-cereal items are more expensive. With non-cereal food items, we also assessed the impact on the sub-category of milk, fruits and their products, which are generally procured after the need for other essential food items is met. Among the non-food items we also assessed the impact on expenditure for medical care and education separately.

We examined variations in implementation of MGNREGS across 19 large states of India with population more than 10 million in the 2011 census. The inequity analysis was restricted to nine less developed states that have been identified by the Government of India for special focus while implementing health programmes [13], which include Assam, Bihar, Chhattisgarh, Jharkhand, Madhya Pradesh, Odisha, Rajasthan, Uttar Pradesh and Uttaranchal The changes in monthly MPCE and the expenditure inequity for food and non-food items across these states were assessed using the consumer expenditure surveys of 2004–05 (pre-MGNREGS) and 2009–10 (post-MGNREGS). Constant prices for 2004–05 were used for the comparisons. Percent change in the inequity ratio from 2004–05 to 2009–10 was computed. A change was defined as statistically significant if the 95% confidence intervals of the estimates for 2004–05 and 2009–10 did not overlap.

### Assessment of consumption expenditure trends in Rajasthan

Rajasthan was the only Indian state where more than 50 percent of the rural households had participated in MGNREGS as estimated from the employment survey 2009–10 data [4]. More detailed analysis of the consumption patterns of the various occupation categories, and the changes from 2004–05 to 2009–10 was therefore done for Rajasthan using data from the two rounds of consumption surveys.

Using the employment survey data for 2009–10, we assessed participation of the various occupation categories in MGNRGES. We estimated the potential contribution of MGNREGS to the rise in MPCE for a household by multiplying the average number of work days availed by that household through MGNREGS in a month with the wage rate for a day’s work under MGNRGES in 2009–10 for Rajasthan (INR 100). This was done for the various categories of occupations. We assumed that the wages earned through MGNRGES were all spent on consumption, as the potential to save from this minimum wage is considered quite limited [14].

## Results

### Implementation of MGNREGS across Indian States

The number of person-days of employment due to MGNREGS estimated from the NSSO employment and unemployment survey was 1.42 billion in India for the 2009–10 financial year, which was half of the 2.84 billion person-days reported by the official programme data (Table 1). The number of person-days of MGNREGS employment reported by the official data for 2012–13 was 2.25 billion, a 20.6% decrease from that reported for 2009–10 by the official data. Interestingly, these official data report a 43.3% decrease in the number of person-days of MGNREGS employment in the nine less developed states from 2009–10 to 2012–13, as compared with a 7.8% increase in the other large ten states of India (Table 1).

**Table 1:** Annual person-days of MGNREGS work estimated from NSSO employment survey and that reported by the official programme data in 19 large states of India, 2009–10 and 2012-13

	Annual number of person-days of MGNREGS work from employment survey 2009–10 (millions)*	**MGNREGS official data†**		
		**2009-10**	**2012-13**	**Percent change in number of person days from 2009–10 to 2012-13**
		**Annual number of person-days generated (millions)**	**Annual number of person-days generated (millions)**	
**Less developed states**				
Assam	27.4	73.3	31.4	−57.1
Bihar	34.0	113.7	90.9	−20.1
Chhattisgarh	65.6	104.2	119.3	14.5
Jharkhand	16.7	84.2	56.5	−32.9
Madhya Pradesh	98.9	262.4	128.5	−51.0
Odisha	42.3	55.4	54.5	−1.6
Rajasthan	350.2	449.8	220.3	−51.0
Uttar Pradesh	120.1	355.9	140.4	−60.6
Uttaranchal	8.6	18.2	18.5	1.2
**Other large states**				
Andhra Pradesh	225.7	404.4	318.0	−21.4
Gujarat	31.3	58.5	28.2	−51.8
Haryana	5.9	5.9	12.9	117.7
Jammu & Kashmir	3.6	12.9	30.4	136.4
Karnataka	18.3	200.3	62.2	−69.0
Kerala	15.2	34.0	83.8	146.6
Maharashtra	18.5	27.4	84.9	209.6
Punjab	4.9	7.7	6.5	−15.5
Tamil Nadu	135.8	239.1	408.1	70.7
West Bengal	92.3	155.2	199.5	28.5
**Total**				
Less developed states	763.7	1517.2	860.2	−43.3
Other large states	551.6	1145.4	1234.4	7.8
India	1422.3	2836.0	2253.2	−20.6

*Computed from NSSO Employment and unemployment survey 2009–10.

†Ministry of Rural Development, Government of India: *NREGA implementation status report for the financial year.* [http://164.100.129.6/netnrega/dash_brd.aspx?fin_year=2012-2013].

According to the NSSO employment survey data, of the rural households in states across India, 34.7% had registered for work under the MGNREGS in 2009–10 (Table 2). This proportion varied from a high of 71% in Rajasthan to a low of 6.6% in Haryana. While 24.2% of the total rural households in India were able to get work under MGNREGS in 2009–10, Rajasthan with 59% and Chhattisgarh with 47.9% were able to provide work for substantial proportion of rural households, while other states had lower coverage. Of the households registered for MGNRGES work, the proportion getting work was variable across the states, with Rajasthan the highest among the less developed states. Overall, in the less developed states, 65.6% of the rural households registered for MGNRGES got work through this programme with an average of 27 person-days per household in a year. The corresponding figures for the other states were 79% of registered households and 23 person-days per household in a year.

**Table 2:** Coverage of MGNREGS across the 19 large states of India, 2009-10*

	**Rural population in millions (2011 census)**	**Percent of total rural households registered for MGNREGS work**	**Percent of registered households that got MGNREGS work**	**Mean MGNREGS work days in a year**		
				**Among all households**	**Among households registering for work**	**Among households that got work**
**Less developed states**						
Assam	26.8	28.6	60.2	6	19	32
Bihar	92.1	17.2	49.6	2	13	24
Chhattisgarh	19.6	58.9	79.8	17	28	35
Jharkhand	25	30.3	49.5	4	12	23
Madhya Pradesh	52.5	68.8	51.7	11	15	29
Odisha	35	40.4	53.5	6	14	26
Rajasthan	51.5	70.9	82.9	42	59	71
Uttar Pradesh	155.1	34.3	75.2	5	24	31
Uttaranchal	7.0	21.1	78.1	6	18	23
**Other large states**						
Andhra Pradesh	56.3	43.4	79.6	16	37	46
Gujarat	34.7	30	59.2	4	15	25
Haryana	16.5	6.6	76.2	2	30	39
Jammu & Kashmir	9.1	19.0	40.0	3	14	32
Karnataka	37.6	15.1	53.0	2	16	30
Kerala	17.5	19.6	55.1	3	14	26
Maharashtra	61.5	13.5	32.8	1	11	33
Punjab	17.3	8.6	59.9	2	18	30
Tamil Nadu	37.2	39.6	83.3	14	36	43
West Bengal	62.2	59.2	72.1	7	12	17
**Total**						
Less developed states	464.6	36.6	65.7	10	27	40
Other large states	349.9	32.1	70.1	7	23	33
India	833.1	34.7	68.3	9	26	37

*Computed from NSSO’s employment and unemployment survey of 2009–10.

**Figure 1:**
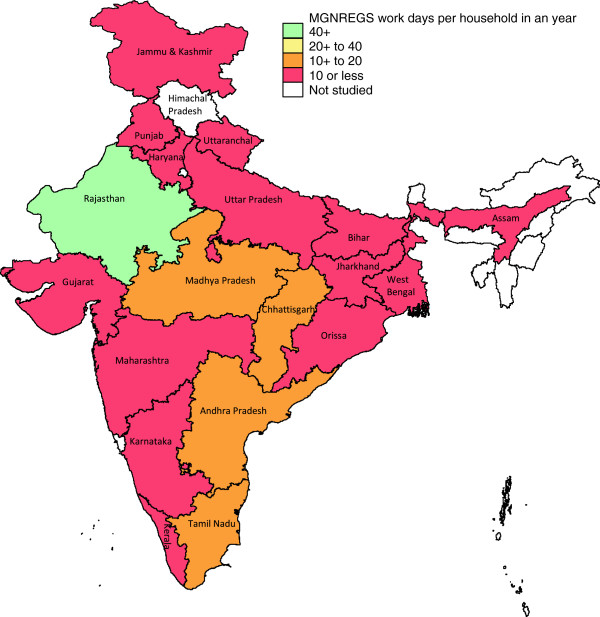
Mean number of MGNREGS work days provided per rural household in a year across the large states in rural India, 2009–10.

Though the objective of MGNREGS is to provide 100 days of unskilled manual labour to households willing to do work in a year, none of the states were close to achieving this target. Rajasthan provided 71 work days on average to the households that got work under MGNREGS, which was the highest, with the next highest being 46 work days on average in Andhra Pradesh. Considering all rural household in each state, the average days of MGNREGS work provided to a household in a year was highest for Rajasthan at 42, with Chhattisgarh a distant second at 17 days; 14 of the 19 large states provided less than 10 days of work on average for each rural household (Table 2, Figure 1). These findings reveal that among the less developed states of India only Rajasthan had close to reasonable coverage of MGNREGS among the rural household in 2009–10.

### Inequities in food and non-food expenditure across less developed states

Table 3 shows the MPCE on food and non-food items across the less developed states of India in 2004–05 and the percent increase to 2009–10 at constant prices among household in the three categories of occupations used in our analysis. The inequity ratios, defined as the ratio of MPCE between the least vulnerable category of households and the most vulnerable category (labourer households), across the less developed states of India in 2004–05 and 2009–10 are presented in Table 4. The highest decline in the inequity ratio for food expenditure was for Rajasthan, a drop of 10.4% from 1.35 to 1.21 with the 95% confidence intervals of these two estimates not overlapping. Smaller relative declines in this inequity ratio from 2004–05 to 2009–10 among four other states were not statistically significant at the 95% confidence level. On the other hand, there were statistically significant increases in the inequity ratio for food in Uttaranchal, Chhattisgarh and Assam. For non-food expenditures, the change in the inequity ratio was not significant at the 95% confidence level in any state except for Uttaranchal where it increased significantly from 2004–05 to 2009–10.

**Table 3:** Monthly per capita expenditure (MPCE) on food and non-food items in 2004–05 and increase in 2009–10 in the less developed states of India*

**Item / State†**	**MPCE by household occupation in 2004–05 in INR (USD)**				**Percent increase in MPCE from 2004–05 to 2009-10‡**			
	**Labourer**	**Self-employed agriculture**	**Other households**	**All households**	**Labourer**	**Self-employed agriculture**	**Other households**	**All households**
**Food items**								
Assam	297 (6.8)	379 (8.7)	371 (8.5)	358 (8.2)	1.0	5.7	14.9	8.0
Bihar	225 (5.2)	305 (7.0)	275 (6.3)	270 (6.2)	8.4	7.7	10.1	5.5
Chhattisgarh	212 (4.9)	258 (5.9)	274 (6.3)	239 (5.5)	2.1	10.6	16.7	3.8
Jharkhand	221 (5.1)	262 (6.0)	304 (7.0)	263 (6.0)	25.0	21.4	19.0	19.5
Madhya Pradesh	185 (4.2)	257 (5.9)	262 (6.0)	232 (5.3)	28.7	37.4	21.6	29.8
Odisha	207 (4.7)	247 (5.7)	297 (6.8)	246 (5.6)	20.8	14.7	13.5	16.0
Rajasthan	261 (6.0)	339 (7.8)	352 (8.1)	324 (7.4)	28.4	15.8	15.0	16.8
Uttar Pradesh	233 (5.3)	302 (6.9)	299 (6.9)	285 (6.5)	13.9	7.8	9.3	8.6
Uttaranchal	278 (6.4)	350 (8.0)	384 (8.8)	346 (7.9)	28.9	21.7	77.1	43.0
**Non-food items**								
Assam	147 (3.4)	181 (4.2)	227 (5.2)	185 (4.2)	22.5	37.7	34.4	35.3
Bihar	109 (2.5)	170 (3.9)	159 (3.6)	147 (3.4)	36.0	36.9	38.1	31.2
Chhattisgarh	149 (3.4)	197 (4.5)	280 (6.4)	186 (4.3)	31.6	31.0	27.0	25.8
Jharkhand	131 (3.0)	155 (3.6)	204 (4.7)	162 (3.7)	53.2	50.1	47.0	46.9
Madhya Pradesh	162 (3.7)	221 (5.1)	266 (6.1)	207 (4.7)	27.0	49.8	43.7	38.4
Odisha	117 (2.7)	144 (3.3)	214 (4.9)	153 (3.5)	47.9	49.6	35.0	43.3
Rajasthan	224 (5.1)	271 (6.2)	300 (6.9)	267 (6.1)	23.7	27.5	31.9	26.1
Uttar Pradesh	182 (4.2)	259 (5.9)	280 (6.4)	247 (5.7)	13.0	8.9	8.6	7.9
Uttaranchal	248 (5.7)	294 (6.7)	352 (8.1)	301 (6.9)	26.5	28.7	300.9	135.0

*Computed from NSSO’s consumer expenditure surveys in 2004–05 and 2009–10.

†Sample size in the 2004–05 survey was 1465 households in Uttaranchal, 3541 in Rajasthan, 7868 in Uttar Pradesh, 4354 in Bihar, 3350 in Assam, 2379 in Jharkhand, 3836 in Orissa, 1997 in Chhattisgarh and 3838 in Madhya Pradesh; sample size in 2009–10 survey was 1048 households in Uttaranchal, 2583 in Rajasthan, 5906 in Uttar Pradesh, 3299 in Bihar, 2616 in Assam, 1758 in Jharkhand, 2975 in Orissa, 1496 in Chhattisgarh and 2731 in Madhya Pradesh.

‡Percent increase based on constant prices for 2004–05.

**Table 4:** Inequity ratio of monthly per capita consumer expenditure of least vulnerable households to most vulnerable labourer households for food and non-food items in less developed states of India, 2004–05 and 2009-10*

	**Inequity ratio for food expenditure**		**Percent change in inequity ratio**	**Inequity ratio for non-food expenditure**		**Percent change in inequity ratio**
	**[95% confidence interval]**			**[95% confidence interval]**		
	**2004-05**	**2009-10**		**2004-05**	**2009-10**	
Assam	1.25	1.42	13.6	1.54	1.69	10
	[1.22-1.29]	[1.35-1.49]		[1.45-1.62]	[1.60-1.78]	
Bihar	1.22	1.24	1.6	1.45	1.48	1.9
	[1.20-1.25]	[1.20-1.29]		[1.39-1.52]	[1.41-1.55]	
Chhattisgarh	1.29	1.48	14.7	1.88	1.82	−2.9
	[1.19-1.39]	[1.41-1.55]		[1.56-2.19]	[1.71-1.92]	
Jharkhand	1.38	1.31	−5.1	1.56	1.49	−4.4
	[1.33-1.42]	[1.24-1.38]		[1.43-1.69]	[1.41-1.58]	
Madhya Pradesh	1.41	1.34	−5	1.64	1.86	13.2
	[1.37-1.45]	[1.25-1.43]		[1.55-1.74]	[1.68-2.03]	
Odisha	1.43	1.35	−5.6	1.83	1.67	−8.9
	[1.39-1.47]	[1.29-1.41]		[1.73-1.94]	[1.59-1.74]	
Rajasthan	1.35	1.21	−10.4	1.34	1.43	6.7
	[1.30-1.39]	[1.15-1.26]		[1.20-1.48]	[1.33-1.53]	
Uttar Pradesh	1.28	1.23	−3.9	1.54	1.48	−3.6
	[1.25-1.28]	[1.18-1.28]		[1.42-1.65]	[1.40-1.55]	
Uttaranchal	1.38	1.9	37.7	1.42	4.49	216.2
	[1.30-1.46]	[1.65-2.14]		[1.13-1.71]	[4.06-4.92]	

*Computed from NSSO’s consumer expenditure surveys in 2004–05 and 2009–10.

The inequity ratios in 2009–10 computed from the consumer expenditure survey and the employment survey were generally consistent between the two surveys for both the food and the non-food items across the less developed states of India (see Additional file 1).

### Consumption expenditure trends in Rajasthan

The overall MPCE by rural household in Rajasthan increased by 21% from 2004–05 to 2009–10 at constant prices (Table 5); the increase was 75.1% at current prices. The increase in MPCE on food items was relatively highest for the most vulnerable labourer households (28.3% at constant prices) as compared with 14.9% for the least vulnerable households. Within food items, this increase was higher for non-cereal food among the most vulnerable as compared with the least vulnerable households. For non-food items, the overall MPCE increase from 2004–05 to 2009–10 at constant prices was 26.3% for rural household in Rajasthan. This increase was somewhat higher among the least vulnerable households (32.1%) versus the most vulnerable households (23.7%). Within the non-food items, the MPCE increase was very high for education, which increased by 82.3% at constant prices for the most vulnerable households and by 140% in the least vulnerable households. Interestingly, the MPCE for medical care decreased by 19.3% at constant prices among the most vulnerable households from 2004–05 to 2009–10 and increase slightly by 3.5% in the least vulnerable households.

**Table 5:** Household monthly per capita consumer expenditure by household occupation in rural Rajasthan, 2004–05 and 2009-10*

**Item**	**MPCE in current prices in 2004–05 by type of household in INR (USD)**				**Percent increase in MPCE from 2004–05 to 2009-10† (in constant prices)**			
	**Labourer**	**Self- employed agriculture**	**Other households**	**All households**	**Labourer**	**Self-employed agriculture**	**Other households**	**All households**
	**(N = 706)**	**(N = 1696)**	**(N = 1139)**	**(N = 3541)**				
All items	485 (11.1)	610 (14.0)	652 (15.0)	591 (13.6)	26.2	20.9	22.8	21.0
All food items	261 (6.0)	339 (7.8)	352 (8.1)	324 (7.4)	28.3	15.7	14.9	16.8
Cereal foods	79 (1.8)	87 (2.0)	89 (2.0)	86 (2.0)	19.0	11.2	15.5	13.3
Non-cereal foods	182 (4.2)	252 (5.8)	264 (6.1)	238 (5.5)	32.4	17.2	14.7	18.1
Milk / fruits and related products‡	71 (1.6)	137 (3.2)	129 (3.0)	120 (2.8)	38.4	10.3	6.8	10.5
All non-food items	224 (5.1)	271 (6.2)	300 (6.9)	267 (6.1)	23.7	27.4	32.1	26.3
Medical care	34 (0.8)	45 (1.0)	33 (0.8)	39 (0.9)	−19.3	−18.8	3.5	−15.0
Education	8 (0.2)	17 (0.4)	20 (0.5)	16 (0.4)	82.3	81.6	140.4	90.0

*Computed from NSSO’s consumer expenditure survey in 2004–05 and 2009–10.

†Sample size in the 2009–10 survey was 760 households for labourer, 951 in self-employed agriculture and 872 in other households.

‡Milk/fruit and related products are a subset of non-cereal foods.

The inequity ratio, defined as the ratio of MPCE between the least vulnerable category of households and the most vulnerable category, reduced for food items significantly by 10.4% from 2004–05 to 2009–10. This decline was mainly due to the reduction in the inequity ratio for non-cereal items which dropped by 13.4% from 1.45 in 2004–05 to 1.26 in 2009–10 which was significant at the 95% confidence level (Table 6), indicating that the inequity in consumption of higher level food items had decreased. Within non-cereal food items the reduction in the inequity ratio for milk and fruit related products was even higher at 22.8%. The change in the inequity ratio for non-food items was not statistically significant at the 95% confidence level. However, for the subset of education, there was a significant increase in the inequity ratio.

**Table 6:** Consumption inequity ratios in rural Rajasthan in 2004–05 and 2009-10*

**Item**	**Inequity ratio (95% confidence interval)**		**Percent change**
	**2004-05 (N = 3541)**	**2009-10 (N = 2583)**	
All items	1.35 (1.27 - 1.42)	1.31 (1.24 - 1.38)	−2.7
All food items	1.35 (1.30 - 1.39)	1.21 (1.15 - 1.26)	−10.4
*Cereal foods*	*1.12 (1.09 - 1.15)*	*1.09 (1.03 - 1.14)*	*−3.0*
*Non cereal food items*	*1.45 (1.39 - 1.51)*	*1.26 (1.19 - 1.32)*	*−13.4*
*Milk/fruit related products*	*1.81 (1.72 - 1.90)*	*1.40 (1.29 - 1.51)*	*−22.8*
All non-food items	1.34 (1.20 - 1.48)	1.43 (1.33 - 1.53)	6.7
*Medical care*	0.99 (0.94 - 1.05)	1.28 (1.00 - 1.56)	28.3
*Education*	1.81 (1.36 - 2.27)	3.30 (2.95 - 3.66)	82.1

*Computed from NSSO’s consumer expenditure survey in 2004–05 and 2009–10.

### Contribution of MGNREGS to MPCE in Rajasthan

In the high implementation state of Rajasthan, 75.2% households among the most vulnerable category of labourers got work under MGNREGS in 2009–10 (Table 7). The mean MGNREGS work days per households per year was more than double among this category of households (56 days) than among the least vulnerable category households (24 days). Among the labourer households, the total MPCE by the households that got MGNREGS work was INR 798 (USD 17) as compared with INR 1021 (USD 22) by households that did not seek MGNREGS work, indicating that the most needy benefited from this scheme.

**Table 7:** Participation in MGNREGS by household occupation in rural Rajasthan, 2009-10*

**Household occupation**	**Percent of households†**	**Percent that got MGNREGS work**	**Mean MGNREGS work days**	
			**All households**	**Beneficiary households**
Labourer	27.6	75.2	56	74
Self-employed agriculture	48.6	60.9	43	69
Other households	23.8	36.3	24	66
All households	100	59.0	42	71

*Computed from NSSO’s employment and unemployment survey 2009–10.

†The sample of labourer households was 716, of self-employed agriculture was 994, and of other households was 872; the percentages shown are adjusted using the distribution of these categories of household in the rural Rajasthan population as the reference.

Considering all households together, MGNREGS was estimated to have resulted in an average increase of 7.2% in the MPCE in rural Rajasthan in 2009–10 (Table 8). This estimated increase in MPCE due to MGNREGS was highest for all labourer households considered together (12.7%). Among the labourer households, the increase in MPCE was 25.9% for those that got 100 or more days of MGNREGS work in a year and was 13.9% for those who got less than 100 days of MGNREGS work in a year.

**Table 8:** Estimated increase in monthly per capita consumer expenditure (MPCE) due to participation in MGNREGS in rural Rajasthan, 2009-10*

**Household occupation**	**Number of households**	**MGNREG work days for household**	**MPCE in INR (USD)**	**Estimated MGNREGS contribution to MPCE in INR (USD)**	**MGNREGS induced increase in MPCE (%)**
Labourer	357	< 100 days	829 (17.8)	101 (2.2)	13.9
	186	≥ 100 days	749 (16.1)	154 (3.3)	25.9
	716	All^†^	836 (17.9)	106 (2.3)	12.7
Self-employed agriculture	379	< 100 days	988 (21.2)	85 (1.8)	9.4
	163	≥ 100 days	909 (19.5)	157 (3.4)	20.8
	994	All^†^	989 (21.2)	68 (1.5)	6.9
Other households	222	< 100 days	1003 (21.5)	82 (1.8)	8.9
	80	≥ 100 days	1127 (24.2)	157 (3.4)	16.2
	872	All^†^	1126 (24.1)	41 (0.9)	3.7
All households	958	< 100 days	942 (20.2)	89 (1.9)	10.4
	429	≥ 100 days	869 (18.6)	155 (3.3)	21.7
	2582	All^†^	981 (21.0)	66 (1.4)	7.2

* Computed from NSSO’s employment and unemployment survey 2009–10.

^†^All includes both beneficiary and non-beneficiary households.

## Discussion

We examined the potential of the public sector national employment guarantee scheme in India, the MGNREGS, which had an expenditure of USD 7.2 billion in the last financial year, to reduce inequities in food and non-food consumption between the most and least vulnerable households in rural India. Detailed analysis of the NSSO survey data from Rajasthan state, which had the highest level of implementation of this programme in 2009–10 with 59% of the rural households having obtained MGNREGS work equivalent to 350 million person-days, revealed that the food consumption inequity between the most and least vulnerable households had dropped by over 10% between the pre- and post-MGNRGES periods. This level of decline in food consumption inequity was not seen in other states with lower levels of MGNRGES implementation, suggesting that MGNRGES likely contributed to this. Although suggestive, it should be noted that our analysis does not allow a firm causal association of this inequity decline with MGNRGES. It is interesting to note that the decline in food consumption inequity in Rajasthan was driven by a 23% decline in inequity for the non-cereal food items between the most and least vulnerable households, suggesting that post-MGNRGES the most vulnerable households were able to spend relatively more on the non-cereal foods making their diet more complete. The overall monthly per capita expenditure ability was estimated to have increased by 26% for the most vulnerable rural households in Rajasthan that got 100 or more days of MGNREGS work that is recommended under this programme.

The NSSO survey data showed that 37% of the rural households in the nine less developed large states of India had registered for work with MGNRGES, suggesting a reasonably high demand. In Rajasthan, 83% of the registered households got MGNRGES work in 2009–10, whereas in the other eight less developed states this figure was 61%. Among the households that got MGNRGES work in these eight states, the average number of person-days of MGNRGES work in a year was 30, which was much lower than the corresponding number of 71 person-days in Rajasthan. While the data from Rajasthan suggest that a higher level of implementation of MGNRGES in the other less developed states of India may reduce food consumption inequities there as well, the decline of 43% according to the programme data in the number of person-days of MGNRGES work provided in 2012–13 in the less developed states of India as compared with 2009–10, raises concerns. It is possible that some or most of this decline reported by the official data may be due to more accurate data reporting in recent years. However, in the absence of the ability to discern this, on face value it seems that the potential benefits of MGNRGES in reducing food consumption inequity have not only not increased since 2009–10, but possibly have decline since then.

For the non-food items, none of the less developed states of India had a significant change in consumption inequity from the pre- to the post-MGNRGES period, except Uttaranchal that a 2-fold increase in this inequity between the most and least vulnerable households. The latter seems to have been due to the 3-fold increase in the per-capita expenditure on non-food items at constant prices by the least vulnerable households on the one hand, and an insignificant impact of MGNREGS on the most vulnerable households on the other hand with a low average of 6 person-days of MGNREGS work per rural household in 2009–10.

In Rajasthan, while the overall non-food consumption inequity between the most and least vulnerable households was not significantly different in the pre- and post-MGNRGES surveys, the inequity in spending on education increased significantly by 82%. However, examination of the absolute spending on education reveals that this was quite low in Rajasthan in 2004–05, 3% of the total expenditure or less for any category of households, and increased in 2009–10 by 82% at constant prices among the most vulnerable households and by 140% among the least vulnerable households, indicating that absolute expenditure on education increased among all types of rural households but increased relatively more among those in the highest socioeconomic group.

The change in medical care expenditure in Rajasthan from 2004–05 to 2009–10 was unique, as among the categories studied it was the only category in which the expenditure decreased at constant prices during this period for all rural households considered together. This decrease was 19% among the most vulnerable households as compared with a 4% increase in the least vulnerable households. One possible explanation for this is that the implementation of the National Rural Health Mission, a broad programme launched in 2005 to improve rural health services in the public sector [15], may have helped decrease the direct expenses on medical care by the most vulnerable households. This possibility is supported by a recent report which has suggested that the utilization of delivery care from public health facilities has increased in less developed states of India during 2004–08, and that the household cost of delivery care has declined for the poor after adjusting for inflation [16]. Another programme aimed at health protection of the poor, the Rashtriya Swastha Bima Yojna or the National Health Insurance Scheme, was launched in April 2008 to provide cashless insurance for hospitalization to households below the poverty line [17]. However, we are unable to assess if this programme could have had an impact on reducing the medical care expenditure by the poor in Rajasthan by 2009–10, though this possibility cannot be ruled out.

Income and wage inequities have been reported to contribute to disparities in health outcomes in more developed countries [18,19]. The data presented in this paper suggest that a higher level implementation of MGNREGS, the livelihood protection scheme for rural households in India, has the potential to reduce food consumption inequities in less developed states. Additional income in low-income households is preferentially allocated for food [20], and therefore it is not surprising that we found reduction in food consumption inequity in Rajasthan where the implementation of MGNREGS was relatively high. It is interesting to note that while nutritional status in India has improved generally over the past two decades, there have been reports suggesting that the nutrition inequalities among women and children have not improved between socioeconomic groups [21,22]. While we did not have data to assess the link between nutritional improvement and better health outcomes in this study, better living conditions including nutrition leading to better health is expected and an important component of the social determinants of health framework [1]. This underscores the potential health benefits from an adequate implementation of the MGNREGS. On the other hand, we did not find significant changes in non-food consumption inequities related to MGNRGES in our analysis. It is therefore unclear if these inequities would benefit widely from higher levels of MGNREGS implementation. The role of governments to facilitate improvements in the broader determinants of health is critical [23]. The widely variable implementation of MGNREGS across the Indian states, and the apparent decrease in coverage in the less developed states over the recent years, suggest that a more firm and consistent policy approach in addressing the broader determinants of health in India is needed.

The analysis presented in this paper has some limitations that must be considered while interpreting the findings. First, our results are suggestive of a beneficial effect of MGNREGS in reducing food consumption inequities in Rajasthan, but a firm causal relation cannot be inferred as there could also have been other reasons contributing to the changes observed in food consumption inequities. Relevant data were not available to assess the possible influence of confounders through statistical models. Second, while estimating the contribution of MNNREGS to increase in MPCE we assumed that all wages earned through MGNRGES were spent on consumption. Data to fully substantiate this assumption are not available, but it seems reasonable to assume that the potential to generate substantial savings from this minimum wage by the low income households would be quite limited. Third, for the inequity analysis we used consumption data from two cross-sectional NSSO consumption surveys, one before and the other after the start of MGNREGS, but data on household participation in MGNREGS were not available from these. We therefore had to use MGNREGS participation data from another NSSO survey, the employment survey, done during the same period as the second consumption survey. Though the consumption surveys have more detailed data on expenditures by households, it was reassuring to note that the inequity ratios for both food and non-food expenditures were comparable in these two types of surveys in 2009–10 as reported in the additional file with this paper. Overall, these limitations are important to remember, but it is unlikely that these pose any significant challenge to the main finding of our analysis, i.e. a likely beneficial effect of MGNREGS on reducing food consumption inequities at a relatively high level of implementation as observed in Rajasthan.

## Conclusion

This report suggests that work provided by the national employment guarantee scheme in India, the MGNREGS, likely contributed to reducing food consumption inequities between the most and least vulnerable households in Rajasthan in 2009–10. We did not find any evidence for the beneficial impact of MGNREGS in reducing non-food consumption inequities up to 2009–10. The level of implementation of MGNREGS in the large less developed states of India, other than Rajasthan, was quite low in 2009–10 as revealed by the NSSO employment survey data. In addition, the official programme data reported a decline of the MGNREGS person-days in the vast majority of the less developed states of India from 2009–10 to 2012–13. These findings together suggest that the potential of the MGNREGS in improving the lives of the poor in India, including mitigation of inequities between the least and most vulnerable households in the food and non-food determinants of health, is not being adequately realized.

## Abbreviations

INR: Indian Rupee; MGNREGS: Mahatma Gandhi national rural employment guarantee scheme; MPCE: Monthly Per-capita consumer expenditure; NSSO: National sample survey organisation.

## Competing interests

The authors declare that they have no competing interests.

## Authors’ contributions

TRD, RD and LD contributed to the initial concept of this paper. TRD and LD developed the detailed design and drafted the manuscript. TRD conducted the statistical analysis. All authors contributed to the interpretation of the findings and agreed with the final version of the manuscript.

## Supplementary Material

Additional file 1: Table S1 Ratio of monthly per capita consumer expenditure between the least and most vulnerable households for food and non- food items in the less developed large states of India in two surveys, 2009-10. Click here for file
