# Supplementary material for: The national employment guarantee scheme and inequities in household spending on food and non-food determinants of health in rural India
Source: Int J Equity Health. 2013 Oct 15;12:84. doi: 10.1186/1475-9276-12-84 (PMC3856602; doi:10.1186/1475-9276-12-84)
Supplement: Additional file 1: Table S1 — Ratio of monthly per capita consumer expenditure between the least and most vulnerable households for food and non- food items in the less developed large states of India in two surveys, 2009-10. [file 1475-9276-12-84-S1.docx]

**Additional File 1**

| **Table:** Ratio of monthly per capita consumer expenditure between the least and most vulnerable households for food and non- food items in the less developed large states of India in two surveys, 2009-10. | | | | | |
| --- | --- | --- | --- | --- | --- |
| **State** | **Mean MGNREGS work days** | **Inequality ratio for expenditure on food items (95% confidence interval)** | | **Inequality ratio for expenditure on non-food items (95% confidence interval)** | |
|  |  | **Consumer expenditure survey*** | **Employment and unemployment survey**† | **Consumer expenditure survey*** | **Employment and unemployment survey**† |
| Assam | 6 | 1.42 (1.35-1.49) | 1.37 (1.31-1.43) | 1.69 (1.60-1.78) | 1.69 (1.62-1.76) |
| Bihar | 2 | 1.24 (1.20-1.29) | 1.17 (1.13-1.21) | 1.48 (1.41-1.55) | 1.27 (1.18-1.36) |
| Chhattisgarh | 17 | 1.48 (1.41-1.55) | 1.44 (1.32-1.56) | 1.82 (1.71-1.92) | 1.71 (1.53-1.89) |
| Jharkhand | 4 | 1.31 (1.24-1.38) | 1.18 (1.12-1.14) | 1.49 (1.41-1.58) | 1.32 (1.22-1.42) |
| Madhya Pradesh | 11 | 1.34 (1.25-1.43) | 1.34 (1.18-1.50) | 1.86 (1.68-2.03) | 1.67 (1.55-1.79) |
| Odisha | 6 | 1.35 (1.29-1.41) | 1.37 (1.33-1.41) | 1.67 (1.59-1.74) | 1.7 (1.60—1.70) |
| Rajasthan | 42 | 1.21 (1.15-1.26) | 1.24 (1.19-1.29) | 1.43 (1.33-1.53) | 1.48 (1.37-1.59) |
| Uttar Pradesh | 5 | 1.23 (1.18-1.28) | 1.15 (1.11-1.19) | 1.48 (1.40-1.55) | 1.32 (1.26-1.38) |
| Uttaranchal | 6 | 1.9 (1.65-2.14) | 1.96 (1.61-2.31) | 4.49 (4.06-4.32) | 4.33 (3.75-4.91) |
| *NSSO consumer expenditure survey, 2009-10  †NSSO employment and unemployment survey, 2009-10 | | | | | |
